# Active Immunization with Extracellular Vesicles Derived from *Staphylococcus aureus* Effectively Protects against Staphylococcal Lung Infections, Mainly via Th1 Cell-Mediated Immunity

**DOI:** 10.1371/journal.pone.0136021

**Published:** 2015-09-02

**Authors:** Seng Jin Choi, Min-Hye Kim, Jinseong Jeon, Oh Youn Kim, Youngwoo Choi, Jihye Seo, Sung-Wook Hong, Won-Hee Lee, Seong Gyu Jeon, Yong Song Gho, Young-Koo Jee, Yoon-Keun Kim

**Affiliations:** 1 Department of Life Sciences, Pohang University of Science and Technology (POSTECH), Pohang, Republic of Korea; 2 Department of Medicine, Ewha Womans University School of Medicine and Ewha Institute of Convergence Medicine, Ewha Womans Medical Center, Seoul, Republic of Korea; 3 Academy of Immunology and Microbiology (AIM), Institute for Basic Science (IBS), Pohang, Republic of Korea; 4 Department of Integrative Biosciences and Biotechnology, Pohang University of Science and Technology (POSTECH), Pohang, Republic of Korea; 5 Department of Internal Medicine, Dankook University College of Medicine, Cheonan, Republic of Korea; Boston University, UNITED STATES

## Abstract

*Staphylococcus aureus* is an important pathogenic bacterium that causes various infectious diseases. Extracellular vesicles (EVs) released from *S*. *aureus* contain bacterial proteins, nucleic acids, and lipids. These EVs can induce immune responses leading to similar symptoms as during staphylococcal infection condition and have the potential as vaccination agent. Here, we show that active immunization (vaccination) with *S*. *aureus*-derived EVs induce adaptive immunity of antibody and T cell responses. In addition, these EVs have the vaccine adjuvant ability to induce protective immunity such as the up-regulation of co-stimulatory molecules and the expression of T cell polarizing cytokines in antigen-presenting cells. Moreover, vaccination with *S*. *aureus* EVs conferred protection against lethality induced by airway challenge with lethal dose of *S*. *aureus* and also pneumonia induced by the administration of sub-lethal dose of *S*. *aureus*. These protective effects were also found in mice that were adoptively transferred with splenic T cells isolated from *S*. *aureus* EV-immunized mice, but not in serum transferred mice. Furthermore, this protective effect of *S*. *aureus* EVs was significantly reduced by the absence of interferon-gamma, but not by the absence of interleukin-17. Together, the study herein suggests that *S*. *aureus* EVs are a novel vaccine candidate against *S*. *aureus* infections, mainly via Th1 cellular response.

## Introduction

*Staphylococcus aureus* is one of the most significant bacteria in human health care. Globally, 10~20% of the population is *S*. *aureus* carrier [1]. *S*. *aureus* infection, in addition to soft tissue or skin infection and sinusitis, is the major cause of pneumonia, osteomyelitis, and sepsis [2]. In addition, *S*. *aureus* is also one of the main causative bacteria of nosocomial infections [3]. Recently, with the increasing burdens by the *S*. *aureus* strains that have resistance toward methicillin, the methicillin-resistant *S*. *aureus* (MRSA) has become prominent in hospitals, together with the community-acquired *S*. *aureus* [4].

Vaccination, or active immunization, is one of the most cost-effective methods for prevention and control of bacterial infectious diseases [5]. Multidrug resistant *S*. *aureus* strains that are ineffectively treated by the current antibiotics are emerging. Therefore, the development of a vaccine against *S*. *aureus* is in fast needs. For effective prevention of bacterial infections, immune responses should be pathogen-specific and be able to induce long-term memory [6].

Extracellular vesicles (EVs) derived from bacteria are lipid bi-layered particles of 20~200 nm in size [7]. Gram-negative bacteria including *Escherichia coli*, *Pseudomonas aeruginosa*, and *Acinetobacter baumanii* produce EVs [7,8,9]. These EVs have pathogenic components or virulence factors. Therefore, EVs derived from Gram-negative bacteria can be used as vaccination agents by inducing both the innate and adaptive immunity [10]. Recently, we found that Gram-positive bacteria, such as *S*. *aureus*, also have the ability to produce EVs [11]. EVs derived from *S*. *aureus* (SEVs) contain peptidoglycan, lipoteichoic acid, and many pathogenic molecules, such as enterotoxin (SEQ), IgG-binding protein (Sbi), and hemolysin [11]. We also found that the skin application of SEVs induces both the antibody and T cell responses [12]. These findings indicate the potential of SEVs as a vaccine for prevention of *S*. *aureus* infection. To test this, we evaluated the effects of SEV immunization concerning pneumonia and mortality due to *S*. *aureus* infection.

## Results

### *In vitro* innate immune response of SEVs

**Fig 1:**
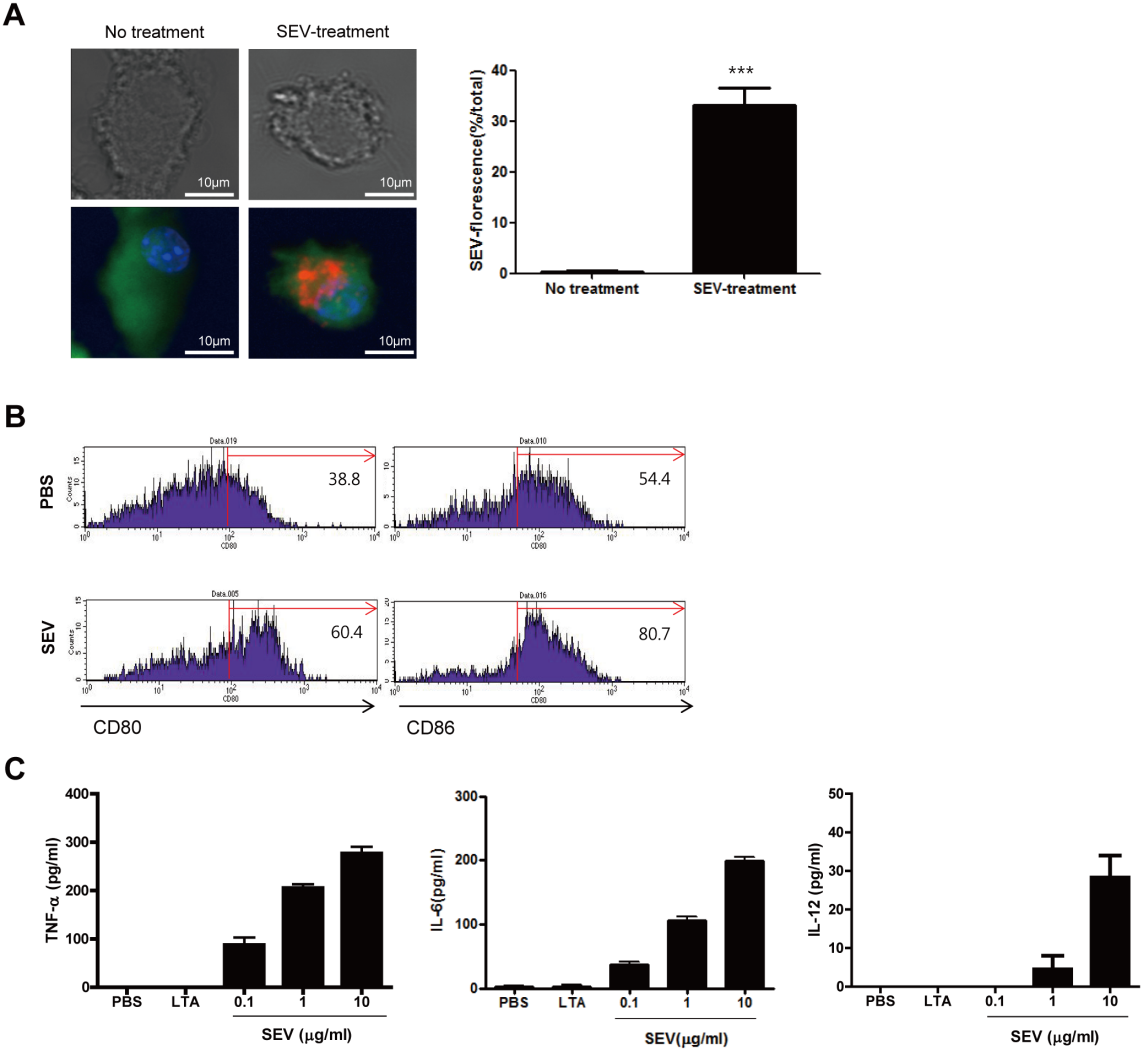
*In vitro* immunogenicity of *S*. *aureus*-derived EVs (SEVs). (A) Uptake of SEVs by bone marrow-derived dendritic cells (BMDCs). BMDCs were treated with SEVs (10 μg/ml) for 24 h. BMDCs cytoplasm were stained with CellTracker Green CMFDA (5-chloromethylfluorescein diacetate, green), nuclei with Hoechst (blue), and SEVs with DiI (red). The quantification of SEV-florescence in no-treatment and SEV-treatment group (n = 20, each group). (B) The expression of co-stimulatory molecules in BMDCs. The expression of CD80 and CD86 in BMDCs were measured 24 h after treatment with SEVs (10 μg/ml) or PBS. (C) Production of pro-inflammatory cytokines from BMDCs 24 h after SEVs treatment. BMDCs were treated with various concentrations of SEVs, and the levels of TNF-ɑ, IL-6, and IL-12 in the cell supernatants were measured by ELISA. *** indicates p< 0.001.

Both the innate immune responses and adaptive immune responses have important roles in clearing infectious bacteria. Dendritic cells (DCs) bridge innate and adaptive immunity via antigen-presentation and the production of cytokines [13]. To assess the immunogenicity of SEVs toward DCs, bone marrow-derived DCs (BMDCs) were treated with SEVs for 24 h prior to the measurement of the expression of co-stimulatory molecules and pro-inflammatory or T cell polarizing cytokines. The uptake of SEVs by BMDCs was observed (Fig 1A). The expression of co-stimulatory molecules such as CD80 and CD86 in BMDCs was enhanced by the treatment with SEVs compared to non-treated control (Fig 1B). In addition, the production of pro-inflammatory mediators, such as tumor necrosis factor-alpha (TNF-α), interleukin 6 (IL-6), and IL-12 from BMDCs were also enhanced by the treatment with SEVs (Fig 1C). These findings suggested that SEVs have the vaccine adjuvant ability to induce adaptive immunity.

### Effect of SEV vaccination against lethality induced by *S*. *aureus* infection

**Fig 2:**
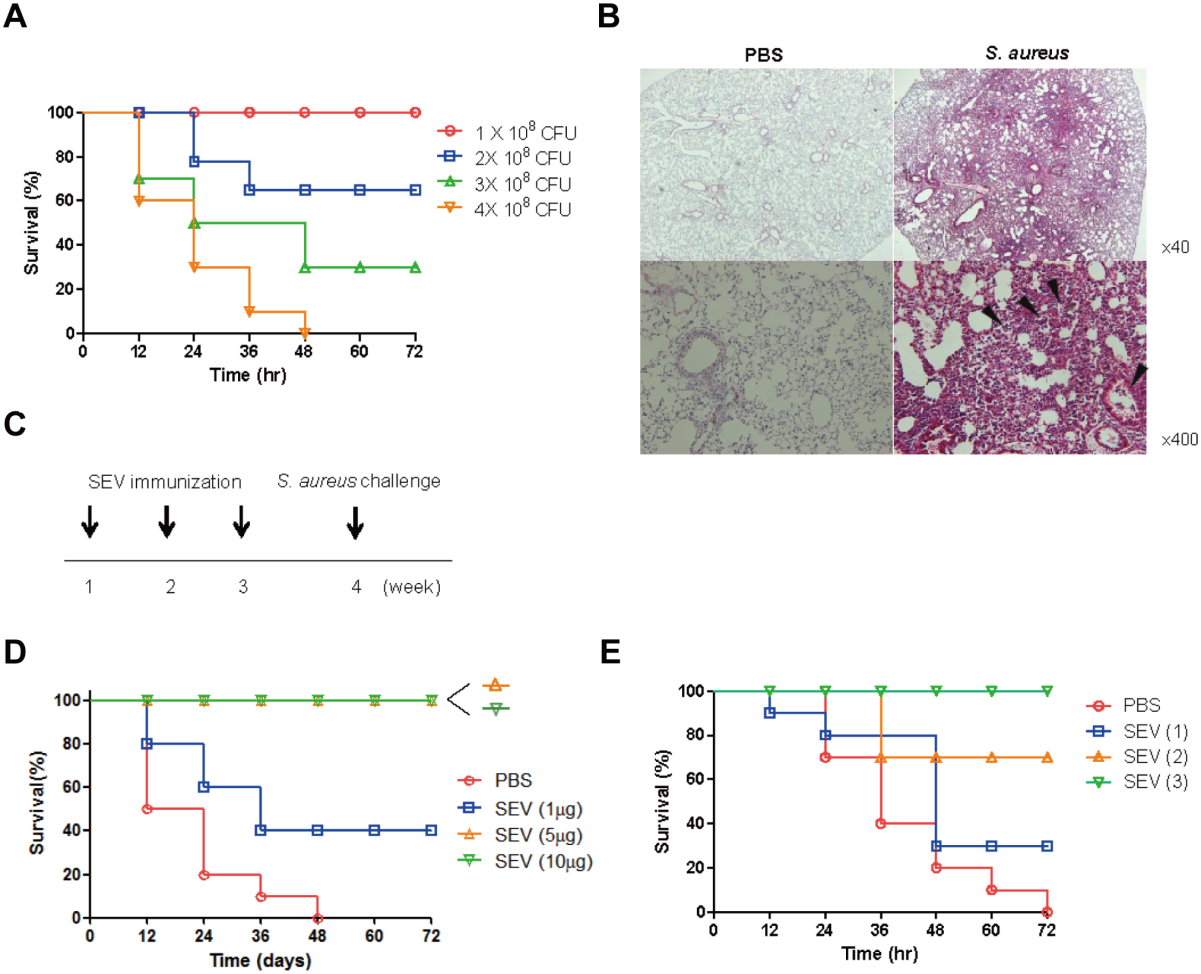
Efficacy of *S*. *aureus* EVs (SEVs) vaccination on protection against lethality induced by *S*. *aureus* lung infection. (A) Determination of lethal and sub-lethal doses of *S*. *aureus* in mouse pneumonia model. Survival rates in mice were evaluated after oropharyngeal application with different doses (1 × 10^8^, 2 × 10^8^, 3 × 10^8^ and 4 × 10^8^ CFU) of *S*. *aureus*. Survival was monitored every 12 h for 3 days (n = 10, each group). (B) Histologic image of mouse lung after oropharyngeal application of *S*. *aureus* (1 × 10^8^ CFU) 24 h post-infection. (C) Study protocol for SEV-immunization and challenge of the lethal dose (4 × 10^8^ CFU*)* of *S*. *aureus*. SEVs and sham (PBS) were injected intramuscularly at weekly intervals for 3 weeks, and then *S*. *aureus* was applied via oropharyngeal route one week after the last immunization. (D) Efficacy of different doses (1, 5, and 10 μg) of SEV vaccination. Survival was monitored every 12 h for 3 days (n = 10, each group). (E) Efficacy of SEV vaccination according to immunization frequency. Survival rates were monitored every 12 h for 3 days in mice immunized with SEVs (5 μg) once, twice, or three times (n = 10, each group).

To test the efficacy of SEV vaccination against *S*. *aureus*-induced pneumonia, mouse pneumonia model was established by oropharyngeal application of different doses of *S*. *aureus* (Fig 2A). The lethal and sub-lethal doses of *S*. *aureus* in this model were 4 × 10^8^ and 1 × 10^8^ colony forming units (CFU), respectively. Histologic findings showed that pneumonia was elicited 24 h after the application of the sub-lethal dose. It was observed that alveolar space and airways were occupied by inflammatory exudates and immune cells (Fig 2B). Based on these results, we evaluated the efficacy of SEVs vaccination on the protection of lethality induced by *S*. *aureus* pneumonia. Mice were immunized intramuscularly with different doses of SEVs three times with an interval of 7 days and then challenged with the lethal dose of *S*. *aureus* 7 days after the last immunization, as shown in Fig 2C. All mice immunized with 5 or 10 μg of SEVs survived, whereas 40% of mice immunized with 1 μg of SEVs and none of the sham-treated mice survived (Fig 2D). Based on these data, we chose 5 μg of SEVs as the vaccination dose for further experiments. We evaluated the vaccination efficacy by the dosing frequency of SEV vaccination. All mice survived after three SEV immunizations, 70% of mice survived after two SEV immunizations, and 30% of mice survived after a single SEV immunization, and none of the sham-immunized mice survived after three sham immunizations (Fig 2E).

### Effect of SEV vaccination on protection against *S*. *aureus*-induced pneumonia

**Fig 3:**
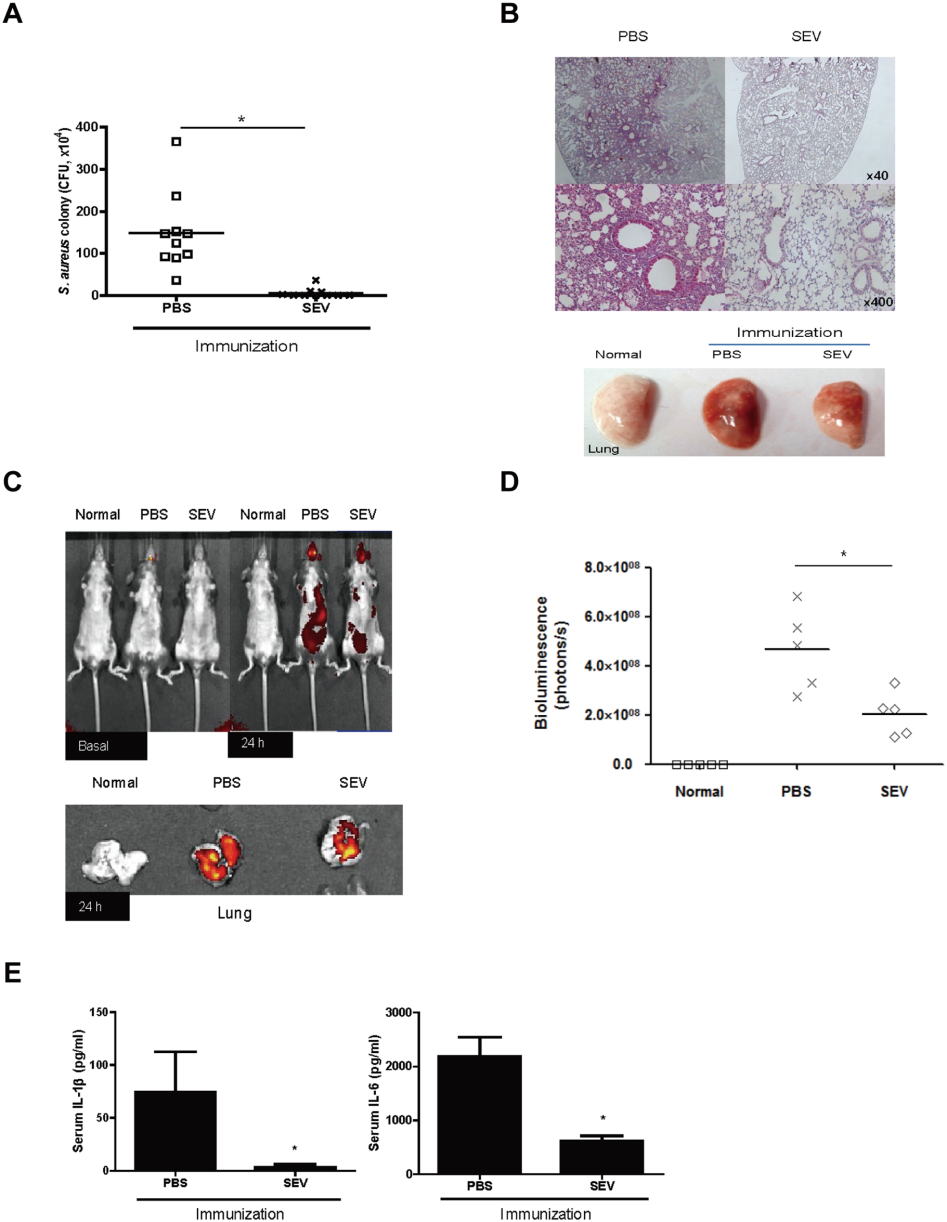
Efficacy of SEV vaccination on protection against pneumonia induced by sub-lethal dose of *S*. *aureus*. For all figures, SEVs (5 μg) and sham (PBS) were injected intramuscularly to mice at weekly intervals for 3 weeks, and then sub-lethal dose (1 × 10^8^ CFU*)* of *S*. *aureus* was applied via the oropharyngeal route one week after the last immunization. Normal: PBS-immunized and PBS-challenged mice; PBS: PBS-immunized and *S*. *aureus*-challenged mice; SEV: SEV-immunized and *S*. *aureus*-challenged mice. (A) Colony forming unit (CFU) counts from lung of SEV- and sham (PBS)-immunized mice 24 h after the *S*. *aureus* challenge (n = 10, each group). (B) Histology (left panel) and gross image (right panel) of lung from SEV- and sham (PBS)-immunized mice after the sub-lethal dose of *S*. *aureus* challenge. (C) Distribution of *S*. *aureus* before and after SEV-immunization. Cy7-labeled *S*. *aureus* was applied via the oropharyngeal route to SEV- and sham (PBS)-immunized mice. Cy7 fluorescence of whole mouse (upper panel) or lung (lower panel) was acquired by IVIS spectrum 24 h after the *S*. *aureus* challenge. (D) Bioluminescence signal in the lung tissue after Cy7-labeled *S*. *aureus* administration. The amount of the bioluminescence signal (photons/s) in the lung tissue was measured by IVIS spectrum 24h after *S*. *aureus* challenge (n = 5, each group). (E) The levels of IL-β and IL-6 in serum of SEV- and sham (PBS)-immunized mice 24 h after the *S*. *aureus* challenge (n = 10, each group). * indicates p< 0.05 vs. PBS.

To assess the efficacy of SEVs vaccine candidate on the protection against *S*. *aureus*-induced pneumonia, mice immunized three times with SEVs (5 μg) or sham injections, were oropharyngeally applied with the sub-lethal dose of *S*. *aureus* (1 × 10^8^ CFU), and were monitored for 24 h after the challenge. The lung tissues of SEV-immunized mice had almost no bacteria burdens, but the lung tissues of sham-immunized mice showed bacterial colonies (Fig 3A). Histologically, inflammatory exudates (pneumonic consolidation) were present in alveolar spaces 24 h after *S*. *aureus* infection only in the tissues isolated from sham-immunized mice (Fig 3B). In addition, *in vivo* imaging system (IVIS) spectrometry analyses showed reduction in the signals for *S*. *aureus* in the whole body and in the lung of mice immunized with SEVs, whereas stronger signals for *S*. *aureus* was detected for sham-immunized mice compared to SEV-immunized mice (Fig 3C and 3D). To evaluate the degree of onset of systemic inflammation in mice with *S*. *aureus*-induced pneumonia, we measured the level of IL-1β and IL-6 in blood as the representative cytokines for systemic induction of sepsis. Significant reductions of both cytokines were detected in SEV-immunized mice compared with sham-immunized mice (Fig 3E). Taken together, these findings indicate an effective vaccination efficacy of SEV immunization against *S*. *aureus* pneumonia.

### Adaptive immunity induced by SEV vaccination

**Fig 4:**
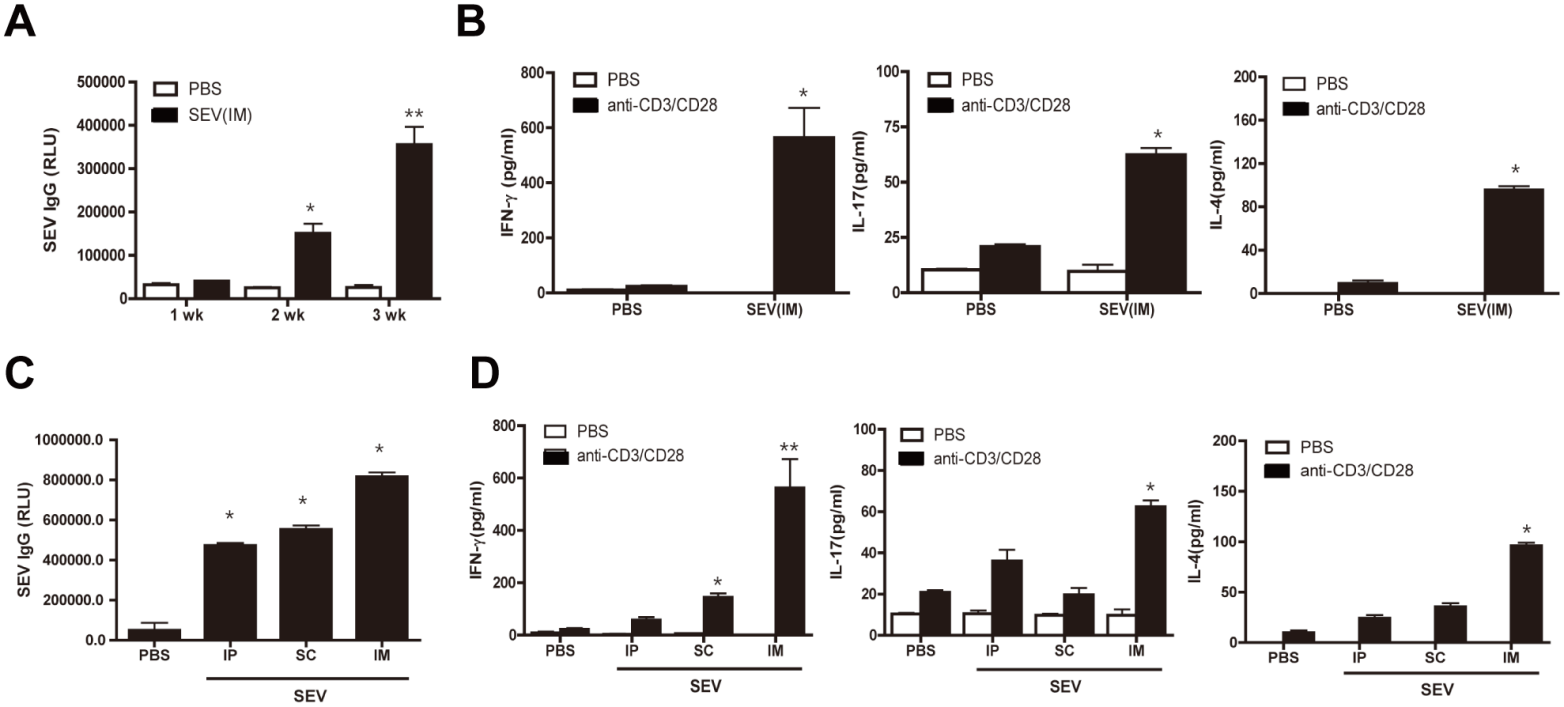
Antibody and T cell responses after SEV vaccination. For (A) and (B), SEVs (5 μg) and sham (PBS) were injected intramuscularly to mice at weekly intervals for 3 weeks (n = 10, each group). (A) The levels of SEV-reactive IgG in serum. Sera were obtained from SEV- and sham-immunized mice 7 days after each immunization and serum levels of SEV-reactive IgG were measured by ELISA. (B) SEV-specific production of IFN-γ, IL-17, and IL-4 from splenic T cells. Splenic T cells were isolated from spleens of SEV- and sham-immunized mice, and then stimulated with anti- CD3/CD28 for 72 h. The levels of IFN-γ, IL-17, and IL-4 in the cell supernatants were measured by ELISA. For (C) and (D) SEVs (5 μg) and sham (PBS) were applied intraperitoneally (IP), subcutaneously (SC), or intramuscularly (IM) at weekly intervals for 3 weeks (n = 10, each group). (C) The levels of SEV-reactive IgG in serum. Sera were obtained from SEV- and sham (PBS)-immunized mice 7 days after the last immunization. (D) SEV-specific production of IFN-γ, IL-17, and IL-4 from splenic T cells. Splenic T cells were isolated from spleen of SEV- and sham (PBS)-immunized mice, and then stimulated with anti-CD3/CD28 for 72 h. The levels of IFN-γ, IL-17 and IL-4 in the cell supernatants were measured by ELISA. * indicates p< 0.05 vs. PBS and ** indicates p< 0.01 vs. the other groups.

We next evaluated the adaptive immune responses induced by SEV immunization. Mice were immunized intramuscularly with SEVs (5 μg) three times. Serum SEV-reactive IgG levels were enhanced after the second immunization and further enhanced after the third immunization compared to sham-immunization (Fig 4A). To evaluate the T cell responses induced by SEV vaccination, mouse spleens were extracted 72 h after the final immunization and then T cells were isolated and re-stimulated by co-incubation with anti-CD3 and anti-CD28 antibodies or PBS. The production of IFN-γ, IL-17, and IL-4 from splenic T cells was significantly increased in SEV-immunized mice after anti-CD3/CD28 stimuli, but these responses were not observed in sham-immunized mice (Fig 4B). Adaptive immunity was evaluated according to the administration routes of SEV vaccine. SEV-reactive antibody production measured 72 h after the third immunization was similar among intraperitoneal, subcutaneous, and intramuscular vaccination routes (Fig 4C). However, the production of IFN-γ, IL-17, and IL-4 from splenic T cells was significantly enhanced by intramuscular injection compared to intraperitoneal and subcutaneous applications (Fig 4D). Intramuscular vaccination with SEV induced Th1, Th17, and Th2 cell as well as IgG antibody responses.

### Role of antibody and CD4^+^ T cells on the protective effect of SEV vaccination

**Fig 5:**
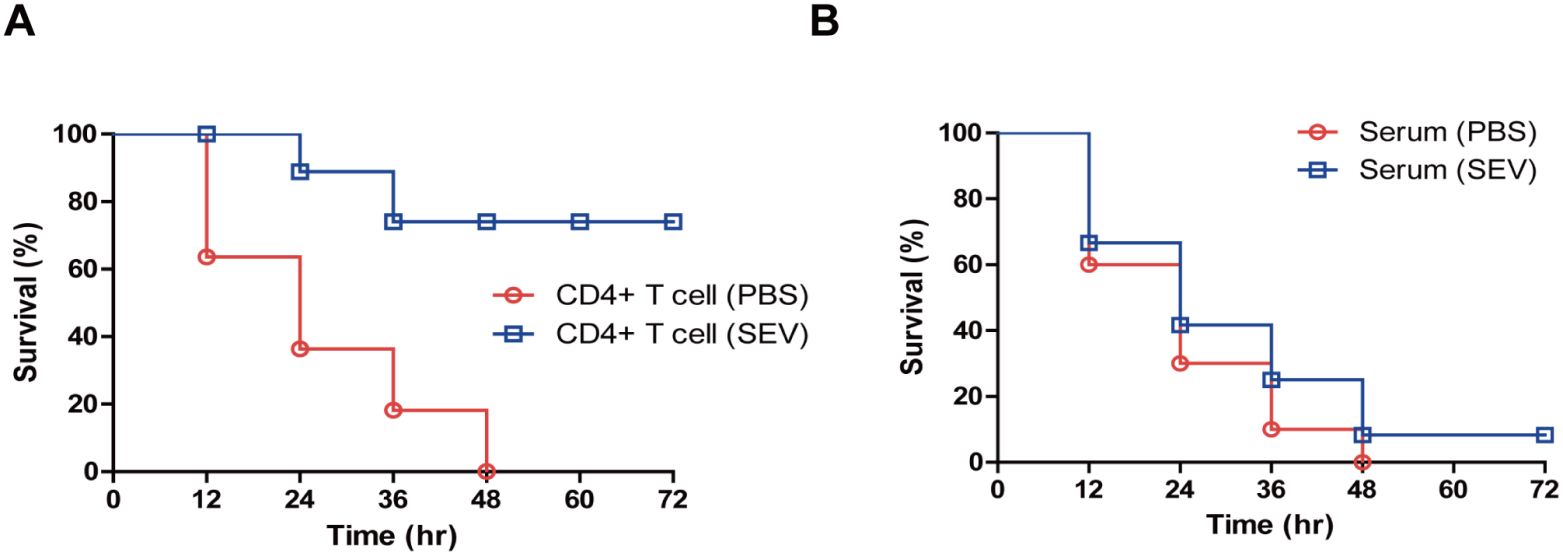
Role of antibody and T helper cells on the protective effect of SEV vaccination. CD4+ T cells and blood sera were isolated from SEV-and sham (PBS)-immunized mice 6 days after three immunizations. Either CD4+ T cells (A) or sera (B) were applied intraperitoneally to naïve mice. After 24 h of the injection, lethal dose (4.0 × 10^8^ CFU*)* of *S*. *aureus* was applied via the oropharyngeal route to the adoptive transferred mice. The survival rates were monitored every 12 h after the bacterial challenge for 3 days (n = 10, each group).

Both the cell-mediated and humoral immune responses are important in providing protective immune responses to the host. To examine whether cell-mediated or humoral immunities are mainly involved in protective responses induced by SEV-immunization, we compared the resistance to *S*. *aureus* infection in naïve mice who received serum or CD4^+^ T cells isolated from the immunized mice. The protocol used for the serum transfer assay was the same as the previous protocol in a study that reported effective anti-bacterial efficacy using passive immunization by serum-transfer [14]. For CD4^+^ T cell transfer, naïve mice received CD4^+^ T cells isolated from SEV-immunized mice. The majority (70%) of the mice that received CD4+ T cells survived the lethal-dose challenge of *S*. *aureus*. None of the mice that received CD4^+^ T cells from sham-immunized mice survived over 48 h after the lethal-dose of *S*. *aureus* challenge (Fig 5A). These results suggested that the adoptively transferred CD4^+^ T cells survived in naïve mice and operated properly. Therefore, mice that received CD4^+^ T cells from SEV-immunized mice showed significant effective protective response against *S*. *aureus* infection compared with the mice that received CD4^+^ T cells from sham-immunized mice. In contrast, no protective ability were observed in both the mice that received serum from SEV-immunized and sham-immunized mice (Fig 5B). These results indicated that the efficacy of vaccination induced by SEV-immunization is mediated mainly by CD4^+^ T cell response, rather than B cell-mediated humoral immune response.

### Role of TLR-mediated signaling on SEV vaccine efficacy

**Fig 6:**
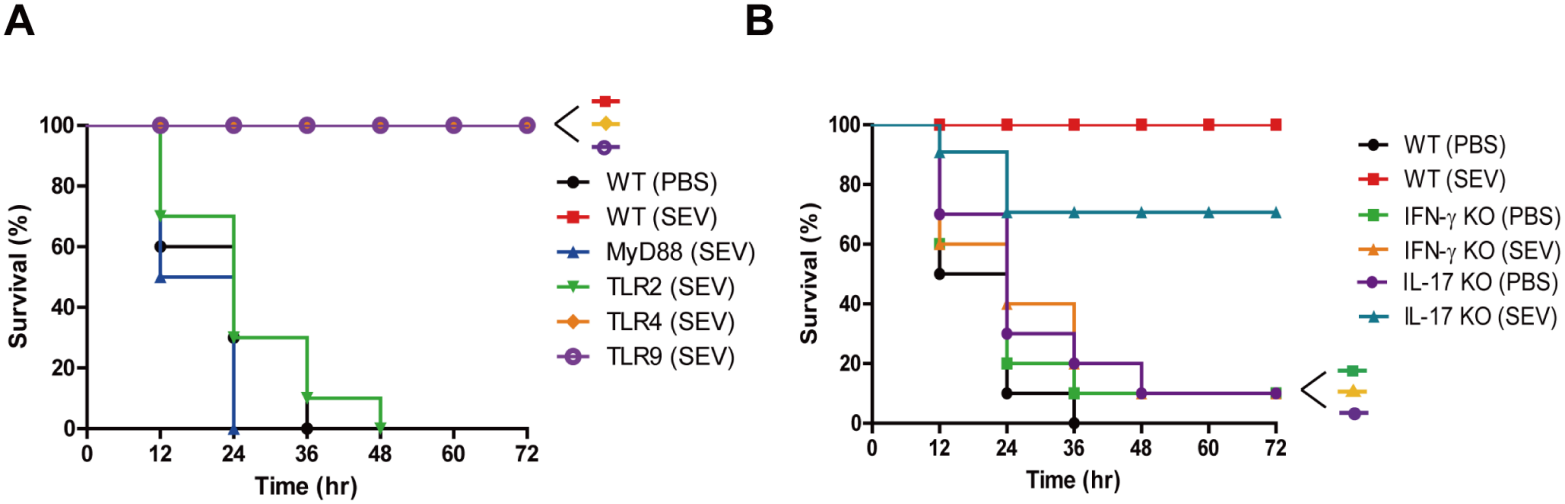
Role of Toll-like receptor signaling and T cell-derived cytokines on the protective effect of SEV vaccination. For all figures, SEVs (5 μg) and sham (PBS) were injected intramuscularly to mice at weekly intervals for 3 weeks, and then the lethal dose (4.0 × 10^8^ CFU*)* of *S*. *aureus* were challenged oropharyngeally to the immunized mice one week after the last immunization. The survival was monitored every 12 h after the bacterial challenge for 3 days (n = 10 each group). (A) The survival rates of wild type (WT), MyD88-deficient, TLR2-deficient, TLR4-deficient, and TLR9-deficient (all C57BL/6 background) mice after *S*. *aureus* challenge. (B) The survival rates of WT, IFN-γ-deficient, and IL-17-deficient (all BABL/c background) mice after *S*. *aureus* challenge.

Both the acquired immunity and innate immunity are important in defense against bacterial infection. In innate immunity, antigen recognition is one of the main steps in the antigen-processing of presentation by antigen presenting cells, including DCs. Toll-like receptor (TLR) signaling pathways, with their down-stream signal of Myd88, are the representative signaling pathways and are divided into nine sub-types. To examine the recognition pathway of SEVs in mice, we evaluated survival rate after SEV-immunization in Myd88^-/-^, TLR2^-/-^, TLR4^-/-^, TLR9^-/-^ and wild type (WT) mice after challenge with the lethal dose of *S*. *aureus*. Like WT mice, TLR4^-/-^ and TLR9^-/-^ mice immunized with SEVs showed 100% survival rate after the challenge. However, all of the sham-immunized and SEV-immunized Myd88^-/-^ and TLR2^-/-^ mice died within 48 h of the challenge (Fig 6A). These results indicate that the recognition of SEVs in the host innate immunity is dependent on the TLR2 signaling, but not on TLR4 and TLR9.

### Role of Th1 and Th17 cytokines on SEV vaccine efficacy

IFN-γ and IL-17 from T cells are important in defense against bacterial infection. To investigate which T helper cells play major roles in SEV vaccination, the survival of IFN-γ^-/-^ and IL-17^-/-^ mice was assessed after SEV-immunization. The SEV-immunized groups of IFN-γ^-/-^ and IL-17^-/-^ mice showed different survival rates after challenge with the lethal dose of *S*. *aureus* (Fig 6B). All the SEV-immunized WT mice survived the bacterial challenge, whereas only 10% and 70% of the SEV-immunized mice survived for IFN-γ^-/-^ and IL-17^-/-^ mice, respectively. All of the sham-immunized WT and knock-out mice died. These data indicated that Th1 mediated cellular response is mainly involved in inducing vaccine efficacy by SEVs immunization, with partial involvement of Th17 mediated cellular response.

### Long-term vaccination effect of SEV immunization for protection against lethality induced by *S*. *aureus* infection

**Fig 7:**
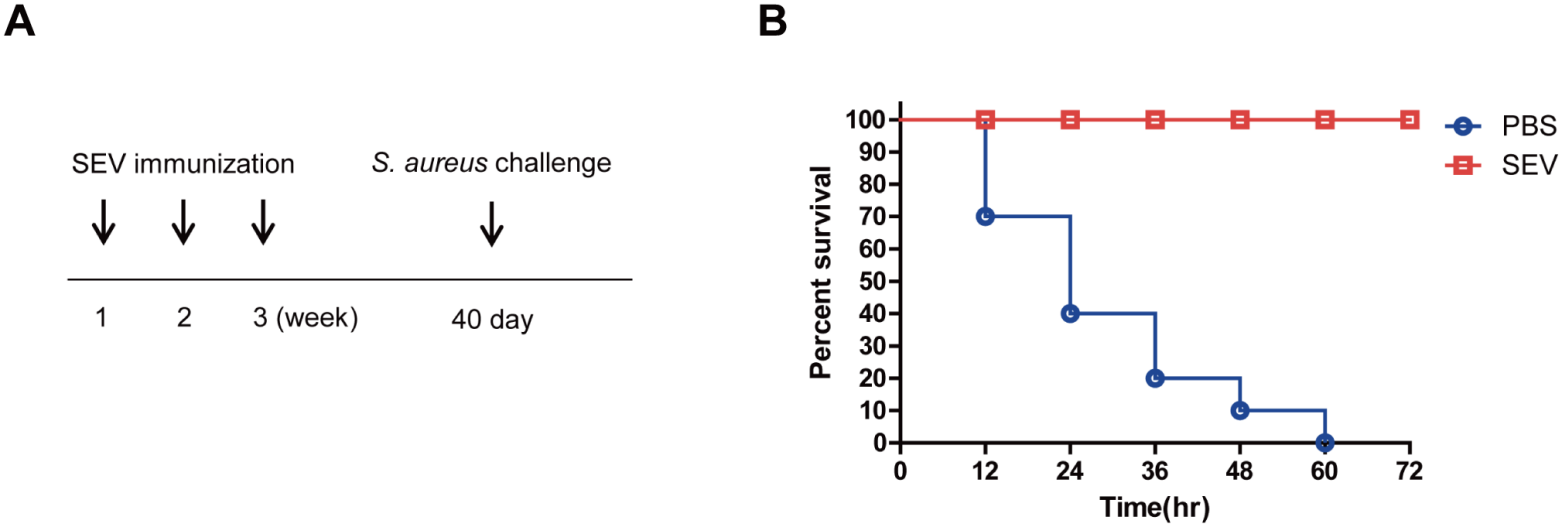
Long-term effect of SEV vaccination on the protection against lethality induced by *S*. *aureus* infection. (A) Study protocol for SEV vaccination and bacterial challenge. SEVs (5 μg) and sham (PBS) were injected intramuscularly at weekly intervals for 3 weeks, and then *S*. *aureus* (4 × 10^8^ CFU) was challenged by oropharyngeal application 40 days after the last immunization. (B) Survival rates of SEV- and sham-immunized mice challenged with *S*. *aureus* (n = 10, each group). Survival was monitored every 12 h for 40 days.

Finally, we evaluated the long-term vaccination effect of SEV immunization for protection against lethality induced by *S*. *aureus* infection. As shown in Fig 7A, SEVs (5 μg) or sham (PBS) were administered intramuscularly to mice at weekly intervals for 3 weeks, then challenged with *S*. *aureus* (4 × 10^8^ CFU) by oropharyngeal application 40 days after the last immunization. As shown in Fig 7B, all the mice that were immunized with SEVs survived the *S*. *aureus* infection, whereas all the sham-immunized mice died. This result suggests that SEV induced protective immune response is maintained in long term.

### Toxicity of SEV vaccination

**Fig 8:**
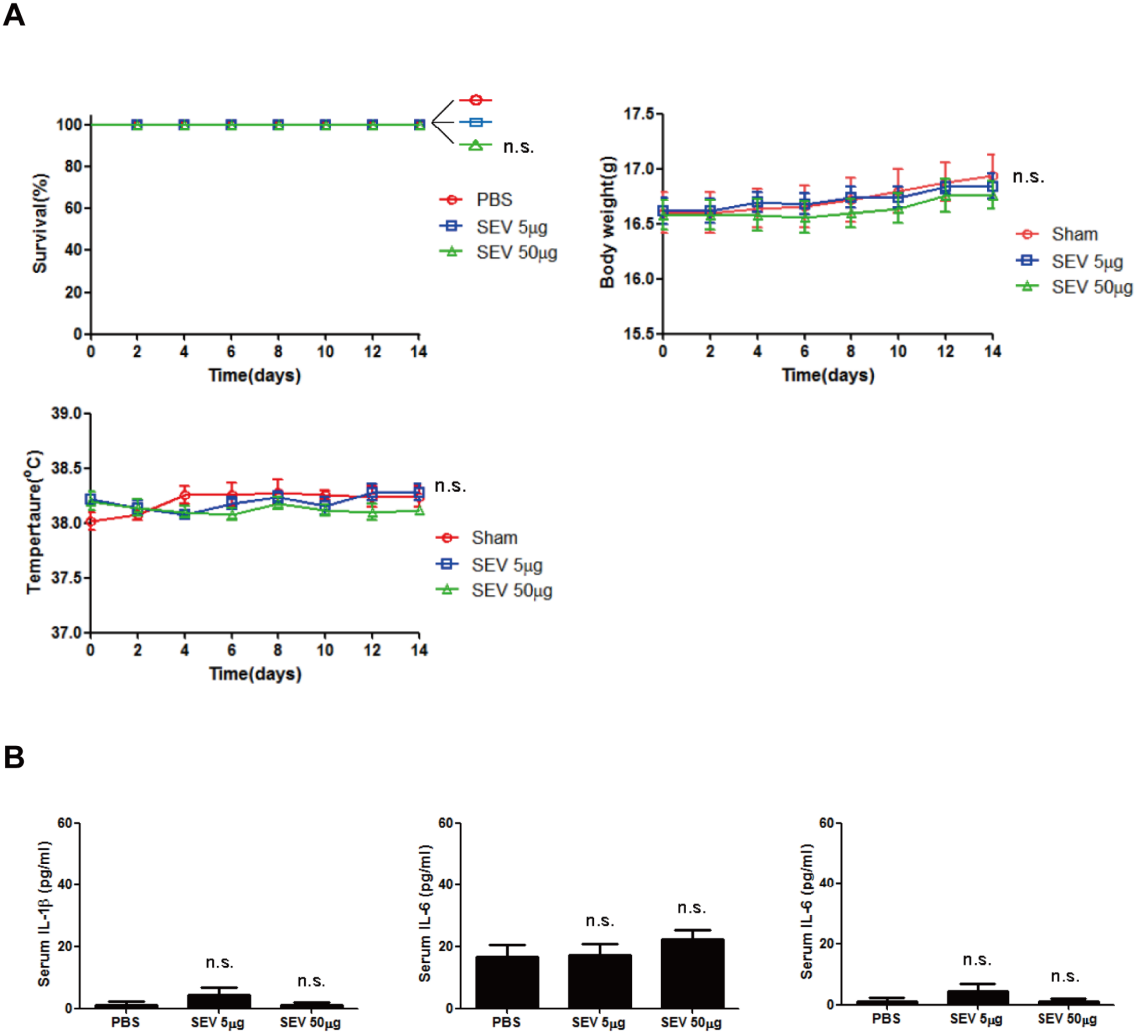
Toxicity of SEV vaccine. Mice were intramuscularly administered with 5 μg or 50 μg of SEV and monitored for 14 days. (n = 10) (A) The survival rate, body temperature, and body weight of mice measured at indicated times. (B) The levels of IL-1β, IL-6 and TNF-α in serum of SEV- and sham (PBS)-immunized mice 24 h after the SEV administration. n.s. indicates not significant.

Safety is the one of the important requirement to be considered when developing and controlling vaccine. Therefore, in order for the SEV vaccine to be developed for use clinically, toxicity is a critical issue that must be assured. To test the toxicity of SEVs immunization, mice were injected intramuscularly with SEVs of the dose used for immunization experiments and of 10 times higher. The survival rate, body temperature, and body weight were monitored for 14 days for examination of any inflammatory responses (Fig 8A). All of the mice survived after SEV administration. Moreover, all the mice injected with SEVs did not show any phenotypical difference compared to the sham-injected mice. In addition, we measured the blood serum cytokines after the SEV administration to evaluate the evidence of systemic inflammation (Fig 8B). As a result, there was no difference in the blood serum cytokine levels between the sham and SEVs administration groups. These results indicate that the administration of SEVs does not cause notable toxic effect on mice.

## Discussion

The hope for an efficacious vaccine against *S*. *aureus* infection has been blunted by the failures of the clinical trials of *S*. *aureus* vaccines [15,16,17]. Multivalent vaccines may work better in humans; previous results have shown that multiple antigen vaccines have better vaccine efficacy than single antigen vaccines *in vivo* [17,18,19]. In addition, as *S*. *aureus* pathogenic and defense mechanisms became better understood, both humoral and T cell-mediated immunities have been revealed to be important in protection against *S*. *aureus* infection [20]. Our present data implicate SEVs as a novel vaccine candidate. In particular, SEVs effectively protect against lethality and pneumonia induced by *S*. *aureus* infection, mainly via IFN-γ-producing Th1 cellular response rather than B cell mediated antibody response.

In our previous study with the SEVs, we have found that SEVs contain membrane proteins, cytosolic proteins, and other virulence factors. Although not fully understood, the pathogenic molecules inside the SEV and EV-associated proteins may induce host immune response differently than secreted proteins. The EV-associated proteins would be protected from antibody-mediated neutralization or protease. Therefore EV-associated proteins could retain longer in the host and travel long-distance. This characteristic, coupled with other physical properties of EVs, like having nano-sized diameters, make EVs ideal for both drug delivery vehicle and immune induction for vaccines [21]. In addition, we have also previously elucidated the vaccination mechanism of EVs using EVs derived from Gram-negative *Escherichia coli* to show that T cell-mediated immunity is the major inducer of protective immune response in mice [10]. Our results in the present study also showed that CD4+ T cell response is more effective as defensive mechanism than B cell-mediated humoral immunity in *S*. *aureus* infection (Fig 5A). These finding demonstrate that T cell-mediated immunity is the major immune response in SEV vaccination.

Studies have sought to develop a vaccine against *S*. *aureus* using capsular polysaccharides or surface proteins as target antigens. However, most failed when applied to human [22,23]. A combinational vaccine of capsular polysaccharides type 5 and 8 (CPS5 and CPS8) originated from Gram-positive *S*. *aureus* was combined with exoprotein A originated from Gram-negative *P*. *aeruginosa*. The combination was used as vaccine agent for human, but it failed in phase III clinical trial. Because CPS8 is expressed in only 40% of *S*. *aureus* strains, the antigen lacks sufficient capacity to prevent wide range of *S*. *aureus* infections [24]. In addition, although CPS5 has proven efficacious in animal models of staphylococcal infection, the use of CPS5 as a target antigen has been hampered by the fact that expression is limited to the stationary phase of *S*. *aureus* growth [25,26]. The other example of failure for *S*. *aureus* vaccine is a trial using a single surface staphylococcal protein, iron surface determinant B (IsdB). Although IsdB is highly conserved and is an important virulence factor, IsdB vaccination showed only partial protective effect against the lethal infection in the mouse model [27]. In addition, passive immunization using antibody specific to single antigen for example, the antibody against ClfA, CP5, CP8, and ABC transporter has also failed [26,28,29]. Combined with the fact that *S*. *aureus* expresses many toxins and immune evasion factors, previous evidence indicates that a single antigen is insufficient for inducing protective immune response. Recently, our proteomic study have demonstrated that SEVs harbors various surface proteins as well as toxins [11]. This prompted the notion that SEVs can be a good vaccine candidate against *S*. *aureus* infection. The present observations that vaccination with SEVs effectively protects lethality and pneumonia induced by *S*. *aureus* infection supports the view that a multivalent vaccine works well for *S*. *aureus* infections.

The previous failures of passive immunization strategies using antibody raise the possibility that B cell-derived humoral responses may not be as important in mediating protection against *S*. *aureus* infection. Previous preclinical results have shown that antibody-mediated responses are important in conferring protection against *S*. *aureus*. For example, passive transfer of antibodies against staphylococcal antigens in animal models conferred protection against *S*. *aureus* infection [30,31]. However, whether antibody-focused vaccine strategies provide any protective effect against *S*. *aureus* infection can be questioned based on the findings from several clinical studies [26,29,32]. Studies have shown the presence of *S*. *aureus* antibodies in most of clinical samples [33,34,35], and colonized subjects have a higher antibody titer against staphylococcal antigens [35]. Moreover, no clinical trial using passive immunization strategies has succeeded [24]. Presently, T cell-mediated immunity rather than antibody response conferred protection against lethality induced by *S aureus* infection. Previous results confirmed that patients with T cell deficiency were highly at risk of staphylococcal infections and B-cell deficient mice were not susceptible to *S*. *aureus* infection than wild type control [36]. These collective findings suggest that T cell-mediated immunity rather than antibody-mediated immunity is critical in the protection against *S*. *aureus* infection.

Th1 and Th17 cell responses, rather than Th2, are considered to have a key role in host defense toward staphylococci infections [19,37]. Animal experiments indicate that IFN-ɤ-deficient mice are more susceptible to *S*. *aureus* infections compared to WT mice [36,38], suggesting that the Th1 cellular response is important in providing protection against *S*. *aureus*. Moreover, IL-17-producing Th17 cells are important for fighting *S*. *aureus* infection by recruiting neutrophils to the site of infection [38]. Furthermore, superoxide-deficient mice were more susceptible to *S*. *aureus* infection [24,39]. These collectively suggest that effective elimination of *S*. *aureus* infections requires both the phagocyte recruitment and phagocytosis, mainly via Th17 cell immunity, and also that the bactericidal activity of phagocytes is mediated mainly by Th1 cell immunity. In the present study, SEV vaccination conferred protection against severe *S*. *aureus* infection, which was mainly dependent on IFN-ɤ rather than IL-17. These findings suggest that Th1 cell-mediated immunity is important in the protection against severe *S*. *aureus* infections, and imply that vaccine development focused on Th1 cell-mediated immunity could provide better vaccine efficacy.

Adjuvants play important roles for promoting antibody production or directing T cells to generate proper cytokines for cellular response [24]. Aluminum hydroxide (alum) and oil-in-water emulsions have been shown to increase antibody titers [40,41,42]. However, increasing studies have shown that T cell response can also be stimulated by the role of adjuvants [42,43,44]. Therefore, using the right adjuvant is important as much as selecting antigens in the vaccine development. SEVs do not require adjuvants since SEVs themselves already contain plethora of bacterial components including various cell wall components[11]. Indeed, the present study showed that SEVs could lead to sufficient innate and adaptive immune responses, without the aid of adjuvants. It is presumed that cell wall components in SEVs may provoke effective immune responses similar to the adjuvants. Peptidoglycan and lipoteichoic acid cell wall components of Gram-positive bacteria induce innate immune responses such as the up-regulation of co-stimulatory molecule expression and Th1/Th17-polarizing cytokine production through TLR2- and Myd88- dependent pathways [45,46,47]. Our results here also have shown that SEVs could enhance the expression of co-stimulatory molecules and the production of IL-12 and IL-6 (Th1 and Th17 polarizing cytokines, respectively) by antigen-presenting cells.

The present study reports an innovative strategy in vaccine development against *S*. *aureus* infections. The vaccine strategy using SEVs provides two main advantages. First, SEVs harbor many bacterial proteins, including cell surface proteins and toxins. Secondly, they do not require adjuvants to elicit an effective adaptive immune response since SEVs themselves can induce both cellular and antibody responses, which confer protection against *S*. *aureus* infections. Collectively, our findings indicate that vaccine development using native EVs from Gram-positive bacteria is a powerful and innovative strategy.

## Material and Methods

### Ethics Statement

This study was carried out in strict accordance with the recommendations in the Guide for the Care and Use of Laboratory Animals of the Pohang University of Science and Technology. The experimental protocols were approved by the Institutional Animal Care and Use Committee at Pohang University of Science and Technology, Pohang, Republic of Korea (Permit Number: 2013-01-0004). All animal experiments were planned to minimize mice suffering.

### Mice

Animal experiments were performed using WT (both C57BL/6 and BALB/c background), TLR2-, TLR4-, TLR9-, and MyD88-deficient (C57BL/6 background), and IFN-γ– and IL-17-deficient (BALB/c background) mice (all 6-week-old females).

### Bacteria strain and culture

For mouse lung infection models, *S*. *aureus* (ATCC 14458) were grown at 37°C in nutrient broth (MERCK 1.05443.0500) until the optical density at 600 nm reached 1.5. Culture aliquots of 500 ml were centrifuged, washed in PBS, and suspended in sterile PBS for mortality studies. Bacteria cultures ranging from 1–4 × 10^8^ CFU were administered in 25 μl of total volume.

### Preparation of SEVs

Overnight bacterial cultures were pelleted at 10,000 g for 20 min, and the supernatant was filtered through a 0.45 μm vacuum filter. The filtrate was concentrated using a QuixStandBenchtop System (Amersham Biosciences), filtered through a 0.22 μm vacuum filter, and was pelleted by ultra-centrifugation in a 45 Ti rotor (Beckman Instruments) at 150,000 g for 3 h at 4°C. The isolated SEV containing pellet was resuspended in PBS and stored at -80°C until use.

### Measurement of SEV-reactive antibody titer

SEV-specific IgG was evaluated in serum harvested from SEV- and sham-immunized mice. SEVs (100 ng) were coated in wells of a 96-well black plate and were blocked with 1% bovine serum albumin in PBS, and loaded with serum samples diluted 500-fold in the 1% suspension. Peroxidase-conjugated anti-mouse IgG was added as secondary antibody and SEV–specific IgG were detected after incubation the addition of a chemiluminescence substrate.

### Adoptive transfer

Serum and spleen were isolated from SEV- and sham-immunized mice one week after the last immunization. For serum transfer experiment, 100 μl serum from either SEV- or sham-immunized mice were intraperitoneally injected in normal naïve mice. For CD4+ T cell transfer, spleens were ground using a cell strainer and were incubated with ammonium chloride solution to lyse red blood cells. The spleen cells were collected from the pellet after centrifugation and were resuspended in RPMI 1640 containing 10% fetal bovine serum and β-mercaptoenthanol (50 μM). Splenocyte T cells were isolated by positive selection using anti-CD3 magnetic bead (Miltenyi Biotec) and CD4+ T cells were serially separated by anti-CD4 microbead positive selection. Cell purity was assessed by flow cytometric analysis. The purified CD4+ T cells (2 × 10^6^ cells in 100 μl PBS) were intravenously injected in naïve mice.

### Immunization protocol and induction of bacterial pneumonia

For SEV immunization, mice were injected intramuscularly three times in weekly intervals in 100 μl PBS. Serum was obtained by eye bleeding 72 h after the last immunization to measure the antibody titers. For the bacteria-induced pneumonia model, mice were administered with *S*. *aureus* via the oropharynx airway. Survival was monitored every 12 h for 6 days. Blood and lung tissues from SEV- and sham-immunized mice were collected 24 h after bacterial challenge for evaluation. To establish bacterial pneumonia in the mouse, anesthetized mice were inoculate by oropharyngeal application with different doses of *S*. *aureus*.

### Mice euthanasia

For humane animal experiment, mice showing severe SIRS indexes were euthanized before death to minimize suffering. After bacterial challenge, mice were monitored every 6 h for 6 days and mice having phenotypical symptoms of systemic inflammatory response syndrome (SIRS) indexes like eye-exudates and piloerection were measured for body temperatures[48,49]. If the body temperatures of the mice dropped below 34°C, mice were euthanized by cervical dislocation. There was no unexpected death in this study.

### Determination of viability as colony forming units (CFU)

A day after the bacteria injection, the bacterial burden in the lung was examined. The lung tissue was homogenized in sterile PBS. The homogenized samples were serially diluted and were plated in nutrient broth agar plates. After overnight incubation at 37°C, CFU were determined.

### Cy-7 labeling of *S*. *aureus*

For labeling *S*. *aureus* with Cy-7, Cy-7 dye (GE Healthcare; PA17101) was-incubated with *S*. *aureus* at 4°C overnight. *S*. *aureus* were pelleted at 10,000 g for 30 min, resuspended in PBS, aliquoted, and stored at -80°C until use. An IVIS spectrometer was used for analysis.

### Measurement of cytokines

The levels of cytokines in fluids and cell-culture supernatants were measured by ELISA in accordance with the manufacturer’s instructions (R&D Systems): IL-1β and IL-6 in serum and IL-1β, IL-6, IL-12p40 and TNF-α in the culture supernatant of DCs, and IFN-γ, IL-17 and IL- 4 in supernatant of isolated splenic T cells and CD4+ T cells.

### Histological analysis

Isolated lungs were fixed in paraffin, sectioned, and stained with hematoxylin and eosin (H&E). Lung tissues were analyzed at 40× and 400× magnification.

### *Ex vivo* studies of isolated DCs

Bone marrow-derived DCs from mice were prepared using a high concentration (20 ng/ml) of granulocyte macrophage colony stimulating factor (R&D Systems). To detect polarizing cytokine from DCs stimulated by SEVs, DCs were exposed to SEVs (5 × 10^5^ per well of a 24-well plate) and incubated for 24 h. After 24 h, the conditioned medium was collected from SEV-treated cells for evaluation of cytokines by ELISA.

### Statistical analyses

For multiple comparisons, one-way analysis of variance (ANOVA) was used first. If significant differences were found, individual t-tests or Wilcoxon’s rank-sum tests were performed between pairs of groups. Differences were considered statistically significant if P < 0.05.
